# Non-Destructive Eggshell Strength Assessment Using Hertz Contact Theory—Part II: Implementation and Validation

**DOI:** 10.3390/foods12061340

**Published:** 2023-03-22

**Authors:** Bart De Ketelaere, Matthias Corion, Ines Adriaens, Paul Van Liedekerke, Wouter Saeys

**Affiliations:** 1 Division of Mechatronics, Biostatistics and Sensors (MeBioS), Department of Biosystems, Katholieke Universiteit Leuven, Kasteelpark Arenberg, 30, 3001 Leuven, Belgium; 2 Division Animal and Human Health Engineering (A2H), Department of Biosystems, Katholieke Universiteit Leuven, Kleinhoefstraat 4, 2440 Geel, Belgium; 3 BIOMATH, Department of Data Analysis and Mathematical Modelling, Ghent University, Coupure Links 653, 9000 Ghent, Belgium

**Keywords:** eggshell strength, Hertz contact theory, fast and non-destructive measurement, low-cost

## Abstract

Eggshell strength is a critical quality factor for consumption eggs as it affects the probability of breakage in practice. In this study, a fast and low-cost methodology for the non-destructive determination of eggshell strength is presented. The method utilized a small steel ball to impact the egg and a microphone to analyse the impact characteristics. Hertz contact theory was applied to relate the measured impact characteristics to the local stiffness of the eggshell. Therefore, a total of 150 eggs were studied on which eight consecutive measurements per egg were taken around the equator at equidistant places. The results showed a strong correlation of 0.93 between the traditional static stiffness measured during quasi-static compression tests and the average stiffness obtained from the new methodology. This paves the way towards fast, low-cost and non-destructive in-line shell strength measurements to reduce the number of cracked eggs reaching the consumer.

## 1. Introduction

Modern egg grading is evolving from a labour-intensive process during which each individual egg is handled by humans towards a highly automated process during which up to 180,000 eggs are sorted per hour. In the sorting process, each egg is individually weighed and inspected for the presence of cracks in the shell, for various types of dirt on the shell and for undesired inclusions such as blood and meat spots [1].

The detection of cracks in the eggshell has been researched by many [2,3,4,5,6], and some of the proposed techniques are commercially available. The proposed techniques use mechanical impact, and either investigate the impulse response of the egg itself or the rebounds of the impactor on the eggshell. A combination of the amplitude of the rebounds and/or the number of rebounds of the impactor is used as an indication of the local mechanical eggshell integrity [7]. An intact eggshell surface allows the impactor to perform several elastic rebounds at high amplitude. In the neighbourhood of a crack, the elasticity of the adjacent shell area is seriously impaired and, hence, the rebound will be heavily damped. By repeating this action at different places on the eggshell surface, a spatial map of the mechanical state of the eggshell can be built. The impact can either be generated by a steel ball embedded in an electromagnetic probe [7], or by the egg itself [8]. As this measuring principle can only reveal local shell quality information, the crack detectors have to test several locations on each egg to obtain satisfactory results. A different approach was suggested by Coucke [3]. Instead of analysing the behaviour of the impactor after excitation, the response of the egg upon impact was considered. When an egg is subjected to a non-destructive impact excitation, the shell will oscillate. Coucke [3] and De Ketelaere et al. [4] observed that eggs with damaged shells show a higher number of resonant peaks than intact eggs. They also showed that, for intact eggs, the impulse response was very similar at every point on the equator, whereas eggs with a damaged shell show a different response at different locations on the equator.

In contrast to the research into the development of crack detection techniques, few studies have aimed at fast techniques to assess the strength of the shell. The main contribution has been made by Coucke [3], who modelled the egg as a mass–spring system and defined a novel eggshell strength parameter named dynamic stiffness, *K_dyn_*. The term dynamic was chosen to stress that the shell strength was measured using dynamic measurements, i.e., after being impacted. Coucke [3] and Coucke et al. [9] reported a correlation of 0.71 between dynamic stiffness and static stiffness, one of the most widely used eggshell strength parameters, for which the measurement procedure is time-consuming. The work by Coucke [3] has been continued in the work of De Ketelaere et al. [1,4], who extended the initial mass–spring model proposed by Coucke [3] to a mass–spring–damper model and showed that the damping of the vibration provides extra information linking the quasi-static measurements to the dynamic measurements.

The economic importance of shell strength information has been discussed by different researchers [10,11,12,13,14,15]. As an important contribution, Mertens et al. [13] demonstrated that shell strength measured at the beginning of the commercial chain (i.e., after laying) was correlated with the probability that the egg would break before it reaches the consumer. As such, grading eggs based on shell strength offers the potential to minimize breakage in practice, for instance by using the weakest eggs for direct breaking and processing, or for local supply only.

In their work, Mertens et al. [16,17] introduced a totally different view on the benefit of the availability of massive quality data of eggs. They show that those data are not only of great use for grading eggs and providing the customer with a high-quality product, but the data also provide interesting information about the health status of the flock that is laying the eggs. By daily monitoring average quality attributes such as egg weight and shell strength, they have demonstrated that health problems in the barn could be traced back. Using concepts of statistical process control, a warning system was devised that produces an alarm when the egg quality is not conforming to the quality of previous days. In that way, problems can be detected even before economical loss occurs.

The above clearly demonstrates the importance of having available fast, non-destructive and reliable measures of egg quality. Given the lack of methodologies aimed at assessing the shell strength in an online way, the aim of this study was to develop a system that has the potential to provide shell strength information on high-speed grading lines. The proposed system involves the analysis of the dynamic impact of a small steel ball on the eggs’ surface, as outlined in De Ketelaere et al. [18]. This approach is fundamentally different from the methods that analyse the vibrations of the egg surface in response to an impact [3,4].

## 2. Materials and Methods

In Part I, we outlined the theoretical background for deriving shell strength information using Hertz contact theory and showed that the dynamic impact of a small, steel ball with an egg can be accurately described by this concept. This study aims to bring the theoretical concept into practice using a setup that allows for automatic, high-speed grading.

### 2.1. Experimental Set-Up

**Figure 1:**
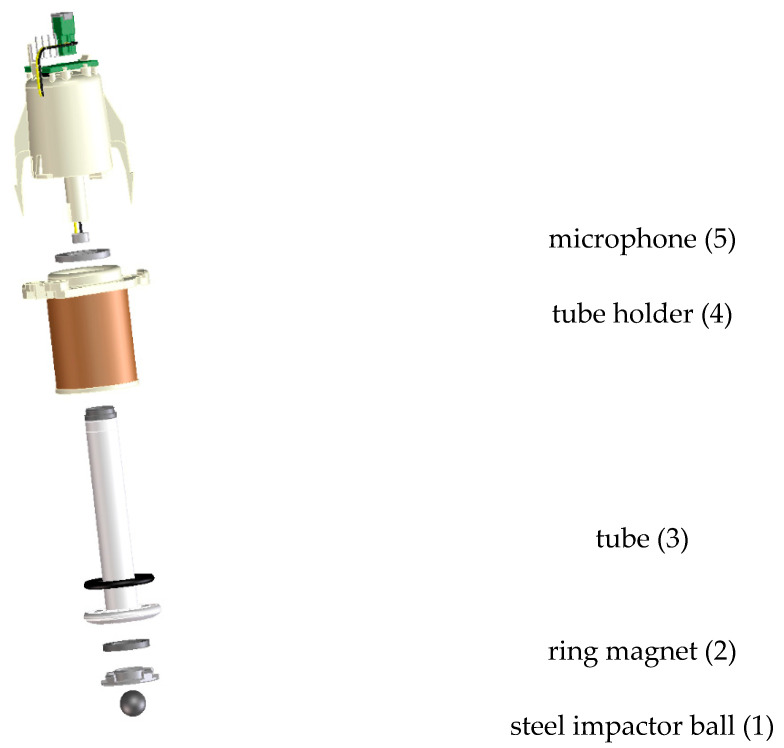
The impactor used for all measurements. It consisted of a plastic tube with a ring magnet at its lower end. Within the magnetic field, a steel impactor ball was positioned. A microphone positioned at the upper end of the probe “listened” to the bouncing of the ball. Picture provided by Moba BV.

For impacting the eggs, a commercial Moba crack detector probe (Moba BV, Barneveld, The Netherlands) was used (Figure 1). It consists of a short plastic tube (3) that was equipped with a ring magnet (2) at its end. A small steel ball (1) is placed in the cavity of the ring magnet. The steel ball has a radius of 4.5 mm and weighed 3 g. The tube can move within a tube holder (4). Typically, the tube holder is fixed above the egg and the tube is then driven towards the egg surface by the magnetic field induced by sending a current through the coil which surrounds the tube holder. When the tube holder approaches the egg, the ball touches the egg and then rebounds several times on the egg surface before coming to rest. A miniature microphone (5; type Primomic EM123, Primo Micro Inc., McKinney, TX, USA) positioned near the end of the plastic tube records the noise generated by the bouncing of the ball at the egg surface. This raw microphone signal is then used for deriving impact characteristics. The microphone signal is digitized using a National Instruments^®^ (Austin, TX, USA) data acquisition board connected to a personal computer at a sampling rate of 50 kHz. Next, the digitized signals are analysed using Matlab^®^ software (Matlab version R2021a, The Mathworks, Inc., Natick, MA, USA).

For relating the microphone signal to the actual impact characteristics that form the basis of Hertz contact theory (see Part I), a set of eggs was not only measured with the set-up described above, but also using a laser vibrometer (Polytec OCV 3001 system; Polytec GmbH, Waldbronn, Germany) as a reference. For details, see De Ketelaere et al. [18]. The eggs that were impacted were supported by two diabolo-shaped rollers, and the probe was directed to the equator of the egg where the actual impact occurred.

### 2.2. Egg and Impact Characteristics

For investigating the relation between impact speed and contact time from microphone data, a first set of 75 fresh, brown-shelled consumption eggs taken from a local warehouse was used. A second set of 150 eggs taken from another supermarket was used to relate classical shell quality parameters—the static stiffness *K_stat_* measured under quasi-static compression and the shell thickness—to the Hertz stiffness *K_H_* obtained from the experimental set-up described in the above section. The egg mass varied between 42 and 64 g; static stiffness varied between 101 and 193 kN/m and shell thickness between 278 and 432 µm.

The static stiffness (*K_stat_*) was measured using a universal tensile and compression test machine (UTS Testsysteme GmBh, Denkendorf, Germany). Eggs were placed horizontally between two flat parallel steel plates and compressed at a speed of 10 mm/min. The resolution of the force sensor was 0.001 N. A maximum force of 10 N was exerted. The force and deformation were recorded throughout the test, and used to calculate the static stiffness as the slope of the force-deformation curve in the range 0.98 to 10 N. The measurement was repeated at three equidistant places on the equator of the egg. The average value of the three measurements was used in the statistical analyses. The egg curvature was determined by taking the egg diameter at the equator (being the impact location, see above), measured by sliding callipers up to an accuracy of 1 mm. Additionally, the weight of the egg was measured with up to 0.1 g accuracy.

Alongside the static stiffness, the dynamic stiffness (*K_dyn_*) of the eggs was also determined based on impulse response measurements. The setup consisted of an impactor rod exciting the egg, and a small microphone recording the vibration of the egg after impact. See De Ketelaere et al. [4] for a detailed description of the setup.

After those non-destructive measurements on the universal tensile and compression test machine and the *K_dyn_* setup, the eggs were placed on diabolo-shaped rollers of the experimental set-up described in Section 2.1. The eggs spun around their long axis while being impacted 8 times by the measurement probe. During such an impact, the small steel ball bounced several times on the egg surface. Typically, around 6 rebounds occurred within a time span of 15 ms. The microphone signal related to the 8 impacts was then analysed on a personal computer. The analysis comprised the following steps:

1. The contact time *τ* (s) was extracted from the microphone signal by quantifying the width of the peaks in the signal. Each bouncing of the ball causes such a peak, and the second peak (i.e., the second time the ball hits the egg) was used;
2. The impact speed *v_n,_*_1_ (m/s) of the second impact (for which the contact time was estimated in the previous point) was estimated by quantifying the time between the first and second impact;
3. The Hertz stiffness *K_H_* (N/m^3/2^) was calculated using Equation (1). For details about the Hertz theory, see De Ketelaere et al. [18].

(1) $$K_{H}={\left(\frac{c}{\tau }\right)}^{5/2}v_{n,1}^{-1/2}µ,$$

where *c* is a constant equalling 3.2145, $v_{n,1}=v_{n1}-v_{n2}$ is the relative speed (m/s) of the impactor and egg, *τ* is the contact time (s), and *μ* is the effective mass of the system (kg):

(2) $$µ=\frac{m_{1}m_{2}}{m_{1}+m_{2}},$$

where *m*_1_ denotes the mass of the impactor (i.e., 3 g) and *m*_2_ the egg mass. The maximal normal deflection *δ_n_^max^* (m) can also be derived as:

(3) $$\delta_{n}^{\max}={\left(\frac{5µv_{n,1}^{2}}{4K_{H}}\right)}^{2/5},$$

from which the maximal force *N_max_* (N) was obtained:

(4) $$N_{max}=K_{H}\delta_{max}^{3/2}.$$

4. For the first set of 75 eggs, those measurements were complemented by measurements taken with the laser vibrometer to have reference values for contact time and impact speed. Since laser measurements are only feasible after demounting the probe, shown in Figure 1, these were taken after all microphone measurements had been collected.

After those measurements, the eggs were broken and the shell thickness was measured at three equidistant locations around the egg equator by means of a micrometre gauge with spherical tips. The resolution of this equipment was 1 µm.

## 3. Results and Discussion

### 3.1. Retrieving Impact Speed and Contact Time from Microphone Data

**Figure 2:**
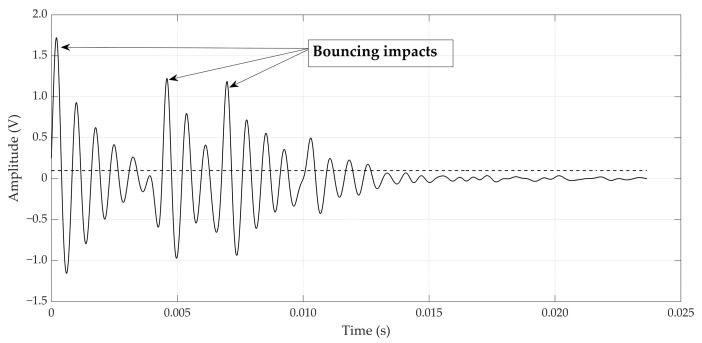
Typical impact data retrieved from the microphone. The dashed line gives the threshold value used to define contact time.

De Ketelaere et al. [18] described a typical laser vibrometer signal resulting from impacting the egg with the given set-up. The time derivative of the speed signal acquired from the laser vibrometer was calculated to obtain the acceleration. Contact time was then calculated from this acceleration signal. The start of the impact was defined as the moment at which the acceleration exceeded 50 m/s^2^, and the end of the impact was similarly defined as the moment at which the acceleration decreased below this limit value of 50 m/s^2^. For the microphone signal, the contact time was retrieved in a similar way. The start of the impact was defined as the moment at which the microphone signal exceeded 0.1 V, and the end of the impact was similarly defined as the moment at which the microphone signal went back below this limit value of 0.1 V. Figure 2 shows a typical impact signal for which there were three consecutive bounces of the impactor ball. As explained in the Materials and Methods section above, the contact time between impactor and eggshell was determined using the second impact, starting at around 5 ms in Figure 2 (middle arrow).

**Figure 3:**
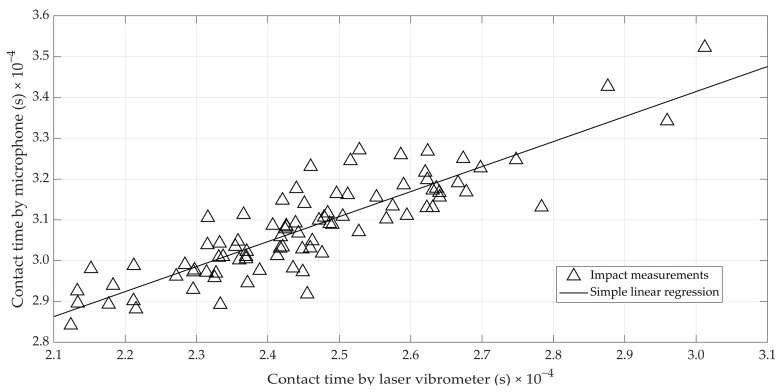
Scatterplot illustrating the relation between the reference contact time as measured by the laser vibrometer, and the contact time derived from the microphone signal.

For the initial set of 75 eggs, utilizing data from both the microphone and laser vibrometer allowed for the determination of a correlation between the reference measurement of the contact time *τ* from the laser vibrometer and the contact time estimated from the microphone data. As explained above, it was only possible to perform both measurements after demounting the probe in Figure 1 and installing the laser vibrometer. Therefore, the correlation obtained was a worst-case correlation since it was not possible to take the laser vibrometer and microphone measurements at exactly the same location. Figure 3 shows a scatterplot relating both contact time estimates for the 75 eggs. It clearly shows a linear relationship between both, and the correlation is rather high (r = 0.87). Despite this relation, the contact time as estimated from the microphone data was systematically higher than the one estimated from the laser vibrometer data. It is hypothesized that the overestimation of the contact time was caused by the dampening of the sound wave resulting from the vibration while it travels from the surface of the impactor ball towards the microphone at the other side of the probe. The relation found is promising, since contact time was shown to have the strongest relation with the reference static stiffness [18], and by using the microphone it is easy to perform multiple measurements per egg (i.e., 8 equidistant measurements around the egg equator were used, see further).

In addition to the contact time between impactor and egg, the impact speed is also of importance (see Equation (1)). One way to determine the impact speed of the second impact of the bouncing ball involves the estimation of the flight time between the first and second impact. However, this would require a solution of the coupled differential equations of the impact situation and the magnetic system. Therefore, we followed an alternative approach by exploiting the correlation between the microphone data and the laser vibrometer measurements.

**Figure 4:**
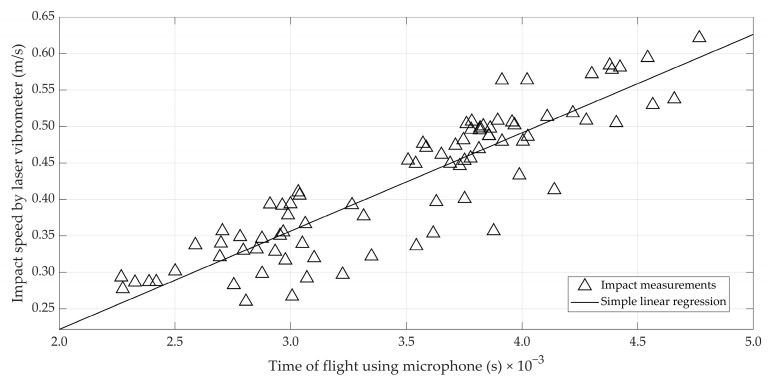
Scatterplot illustrating the relation between the time of flight of the bouncing ball estimated from the microphone signal and the impact speed measured with the laser vibrometer.

For the first set of 75 eggs, a correlation was observed between the impact speed of the second bounce *v_n_*_1_ measured by the laser vibrometer and the time of flight Δ*t* (s) of the bouncing ball estimated from the microphone data. This relation is illustrated in Figure 4. It should be noted, again, that measurements with the laser vibrometer and the microphone had to be performed sequentially, inevitably introducing error. A high positive correlation was found between both parameters (r = 0.89), which allows the estimation of the impact speed *v_n,_*_1_ (m/s) from microphone data using the following formula:

(5) $$v_{n,1}=260.4∆t-0.428.$$

From the above, we can conclude that the impact speed and the contact time between the impactor ball and the egg can be estimated from the microphone data. As these parameters are known to be important factors in relation to shell strength [18], their potential to replace the current, time-consuming practice of measuring the static stiffness under quasi-static compression will be investigated in the next section.

### 3.2. Predicting Shell Strength Parameters Using Microphone Data

For predicting shell quality parameters (static stiffness *K_stat_* and shell thickness), the second dataset of 150 eggs was used. For each impact with the probe, the impact speed was estimated using Equation (5) and the contact time was estimated as illustrated in Figure 2. For all analyses that follow, the average value of the eight impacts around the equator was used. For *K_stat_*, the average of the three quasi-static measurements was used.

**Figure 5:**
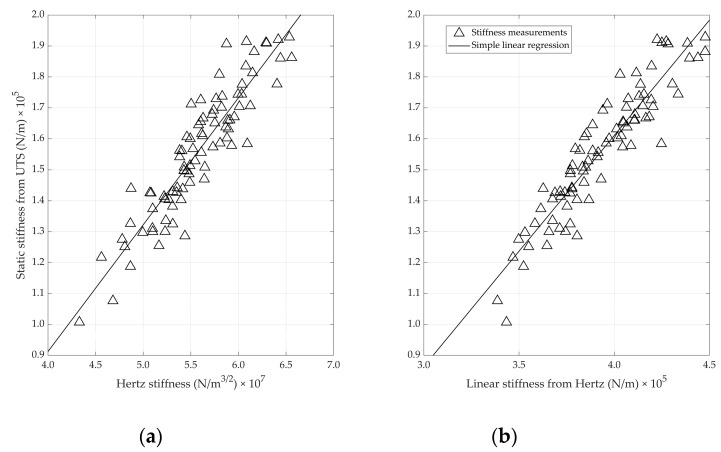
(**a**) Relation between the static stiffness measured during quasi-static compression and Hertz stiffness, (**b**) and a linear stiffness derived from impact measurements.

First, the relation between the static stiffness *K_stat_* and the Hertz stiffness *K_H_* defined in Equation (1) was investigated (Figure 5). The relation between both parameters is strong (r = 0.91), and outperforms the relation found in De Ketelaere et al. [18], who used the laser vibrometer signal instead of a microphone. A possible explanation for this improved correlation is the use of eight measurements around the equator. This strong relation indicates that the proposed fast test set-up can be used as alternative for the time-consuming quasi-static compression test, which is still considered one of the industry standards, without much loss of precision. Furthermore, the results obtained in this study allow the measurement of shell strength in an in-line setting, potentially changing the way eggs are graded. Whereas batches of eggs are classically judged based on their average shell strength assessed using a small sample of eggs, our results open the door towards sorting on the individual egg level. Mertens et al. [13] showed that the strength of individual eggs correlates with the probability of an egg breaking when it travels from producer to consumer, further motivating the use of in-line sorting. Weak eggs could be used for breaking or local delivery, whilst the strongest eggs could be transported on longer distances or could be sold as “premium” eggs.

Measuring the shell strength of all individual eggs also provides the most accurate estimate of the batch average quality. This, in turn, has added value when monitoring flocks of laying hens. Mertens et al. [16,17] showed that daily, accurate information about egg(shell) quality allows for the automated detection of diseases and stress in the henhouse, and the number of eggs tested determines the power of the detection scheme.

In analogy with De Ketelaere et al. [18], linear stiffness was also retrieved from the impact data in this study. It is computed by taking the ratio between *N_max_* in Equation (4) and *δ_n_^max^* in Equation (3). The correlation of this linear stiffness with the static stiffness is marginally stronger (r = 0.93) than the relation *K_H_* versus *K_stat_*, confirming the results reported by De Ketelaere et al. [18]. It should be noted that the stiffness values obtained by impact testing were higher than those obtained by static compression. This can be explained by the different types of measurements, being quasi-static compression versus dynamic (impact).

De Ketelaere et al. [18] showed that the contact time between the impactor ball and the egg is the most important impact-related parameter when the impact speed is higher than 0.2 m/s. Furthermore, in the case of a substantial difference in masses of the two colliding bodies (here *m*_1_ << *m*_2_) it is only the mass of the smallest body that is important. In the considered case, the impactor ball has a mass of 3 g only, limiting the influence of the actual egg mass. Considering two extreme eggs, one egg of 45 and one of 80 g, plugging these masses into Equation (2) gives effective masses *μ* of 2.81 and 2.89 g, respectively. Therefore, the impact of the egg mass is, indeed, limited (<3%).

**Figure 6:**
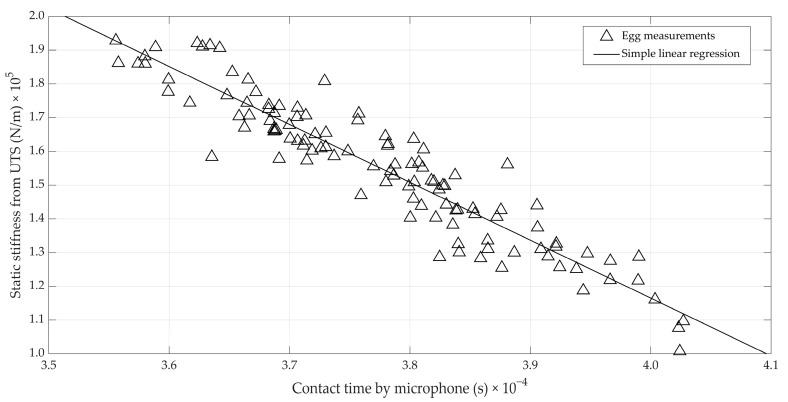
Relation between the static stiffness measured during quasi-static compression and the contact time between impactor and ball, measured by the microphone.

Given the limited importance of the impact speed and egg mass, the relation between the contact time and the reference stiffness *K_stat_* was also investigated. It can be observed from Figure 6 that this relation (r = −0.94) is equally as strong as the relation between *K_H_* and *K_stat_*. The sustained high correlation by neglecting the impact speed and the egg mass may be attributed to the relatively high uncertainty in the estimation of the impact speed. This could be seen from Figure 4, where the impact speed accurately measured by the laser vibrometer was related to the time of flight and, thus, speed. In other words, the loss of information by neglecting impact speed is comparable to the error introduced by estimating impact speed from the time of flight. This suggests that there is still room for improvement.

**Figure 7:**
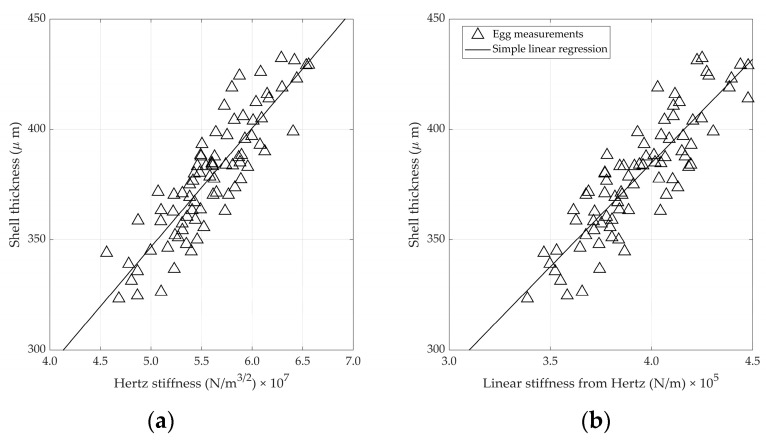
(**a**) Relation between the shell thickness and Hertz stiffness and (**b**) a linear stiffness derived from impact measurements.

In addition to the static stiffness, the thickness of the eggshell is frequently used as an indicator for eggshell strength [1,19]. Strong correlations between *K_stat_* and the shell thickness have been reported, which were confirmed in this experiment (r = 0.86). Hence, a high correlation between the shell thickness and the stiffnesses derived from the impact test was also expected. Figure 7 shows these relations. The correlation between shell thickness and *K_H_* equals 0.88, whereas the correlation between shell thickness and the linear stiffness derived from the impact measurements equals 0.87.

The correlations between the classical stiffness *K_stat_* and the newly defined stiffnesses (*K_H_* or its linear version) are high (r > 0.9). In the study of vibration measurements of eggs after impact, Coucke [3] and De Ketelaere et al. [1,20] reported correlations between the so-called dynamic stiffness *K_dyn_* and *K_stat_*, all ranging from 0.65 to 0.85. The high correlation found in our study might be mainly due to two different reasons. First, the eight replicates taken for each egg lower the standard error on the measured value per egg. Second, the measurement principles behind the dynamic stiffness and the Hertz-related stiffnesses are completely different. Whereas the dynamic stiffness investigates the vibration behaviour of the egg in response to the impact, the Hertz theory investigates the impact characteristics themselves rather than the response of the egg after being impacted.

**Figure 8:**
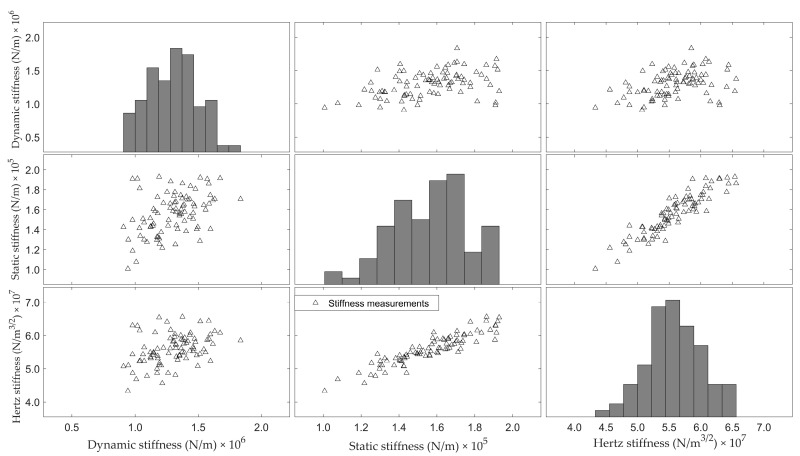
Relation between the static stiffness measured during quasi-static compression and Hertz stiffness (**left**), and a linear stiffness derived from impact measurements.

Figure 8 shows the scatterplot matrix for *K_dyn_*, *K_stat_* and *K_H_*. In the diagonal sub-figures, the histograms of the three parameters are shown. The off-diagonal sub-figures show the two-dimensional scatterplots of the considered parameters. The correlation between the Hertz stiffness and the dynamic stiffness *K_dyn_* is weak (r = 0.39). The correlation between *K_dyn_* and *K_stat_* is also low (r = 0.43). These correlations found are considerably lower than those reported by other researchers [9]. This might be attributed to the fact that the eggs used were mostly originating from young hens. De Ketelaere et al. [1] pointed out that, especially for fresh eggs from young hens, the damping of the vibration of the egg after being impacted is a crucial factor when relating *K_dyn_* to *K_stat_*.

As we observed much stronger correlations between the proposed, novel shell strength indices (*K_H_* and its linearized version) and the classical stiffness (*K_stat_*), it would be interesting to investigate this relation more in depth. This could allow the gaining of more insight into the differences, and whether the *K_H_* provides additional information about the shell quality and its relation to breakage in practice. If that would be the case, *K_H_* could be used as an additional parameter for breeding companies.

## 4. Conclusions

A low-cost, yet accurate, fast and non-destructive measurement principle was demonstrated yielding accurate eggshell strength information. It uses a small impactor and a microphone as main components. Hertz theory was applied to define the most important impact characteristics. It was first demonstrated that the microphone signal was able to provide an estimate of the contact time between impactor and egg. We also demonstrated that the egg mass itself had only a minor influence thanks to the use of a low-mass impactor. Moreover, the need for an accurate estimation of the impact speed could be avoided by using relatively low impact speeds.

The obtained Hertz stiffness was shown to be strongly related to classical shell strength parameters such as static stiffness during quasi-static compression and shell thickness. With the above conclusions about the most dominant impact characteristics, it was also demonstrated that using only the contact time between the impactor and the egg provided an accurate estimate of shell strength. This makes the system attractive from a data processing point of view.

Its fast character and suitability for in-line measurements make it a promising tool to include shell strength measurements during egg grading. This would allow packing stations to use weak eggs for breaking or local delivery, while the strongest eggs could be transported longer distances or could be sold as “premium” eggs. Furthermore, the acquisition of massive amounts of shell strength data opens opportunities for automated detection of problems in henhouses, as shell quality is influenced by the health status of the hens.

## Data Availability

The data presented in this study are available on request from the corresponding author. The data are not publicly available for company proprietary reasons.
